# Root Fractures in the Primary Teeth and Their Management: A Scoping Review

**DOI:** 10.3390/dj10050074

**Published:** 2022-05-01

**Authors:** Enrico Spinas, Gianni Di Giorgio, Martina Salvatorina Murgia, Valentino Garau, Mara Pinna, Nicoletta Zerman

**Affiliations:** 1 Department of Surgical Sciences, Traumatology and Sport Dental Research Center, University of Cagliari, Via Ospedale, 09124 Cagliari, Italy; 2 Department of Oral and Maxillofacial Sciences, “Sapienza” University of Rome, Via Caserta, 6, 00161 Rome, Italy; gianni.digiorgio@uniroma1.it; 3 Department of Surgical Sciences, University of Cagliari, Via Ospedale, 09124 Cagliari, Italy; garauva@yahoo.it (V.G.); mme.pinna@gmail.com (M.P.); 4 Department of Surgery, Dentistry, Paediatrics and Gynaecology, University of Verona, 37134 Verona, Italy; nicoletta.zerman@univr.it

**Keywords:** pulp injuries, children, dental traumatology, orthodontic splint, primary teeth, primary root fracture, dental trauma

## Abstract

(1) Background: Traumatic dental injuries constitute a major global health problem. Primary deciduous teeth of the upper frontal group are frequently affected by trauma, especially at an early age. It is important to treat primary traumatic injuries because early tooth loss can lead to aesthetic and functional alterations. The most common injuries are extrusion, lateral luxation, and intrusion. Root fracture is a less common complication that can lead to tooth extraction if not properly diagnosed and managed. However, there are a lack of data regarding primary root fracture treatment. The literature was reviewed to study the current knowledge on the treatment of these injuries, and to propose an operative protocol based on the results obtained. (2) Methods: A literature search was performed on Web of Science, PubMed/MEDLINE, and SCOPUS. The research focused on the following features: age of the patient; localization of the root fracture and type of displacement suffered (intrusive, extrusive, or lateral); type of emergency treatment or diagnostic test performed and their compliance with IADT guidelines; follow-up duration. (2) Results: Only 8 articles fully met the inclusion criteria, with a total of 46 patients and 62 root fractures. Out of a total of 62 root fractures, regarding only upper incisors, the most common treatment was splinting (*n* = 39) for a period ranging from 3 weeks to 3 months (with an average of six weeks). No treatment was performed for 23 of the root fractures. The splinting performed in most of the included cases was semi-rigid, with the splint held in place using a composite resin material. An orthodontic splint using brackets and 0.5 mm stainless steel wire was used in only in one study. (4) Conclusions: We deduced that the root fracture of primary teeth is a rare traumatic dental injury that can cause numerous complications, such as eruptive problems in the permanent teeth. Correct radiological diagnosis, immediate repositioning and semi-rigid splinting could be conservative methods to prevent premature tooth loss in very young patients.

## 1. Introduction

Primary dentition is, first, essential for functional purposes, as it drives the eruption of the permanent teeth, and secondly, for aesthetic reasons [1]. Numerous studies show how traumatic dental injuries (TDIs) negatively affect the quality of life of young patients and their families [2,3,4]. Several epidemiological studies have shown a high incidence and prevalence of TDI, around 50% [5,6], as confirmed by a recent meta-analysis [7]. Therefore, TDIs constitute a major global health problem. In addition, TDIs to primary dentition can cause long-term consequences to the permanent teeth [8]. The damage to the permanent germs is related to the proximity of the roots of the maxillary deciduous incisors, so the greatest risk occurs in the first two years of children’s lives [9]. In most cases, TDIs to primary dentition are not intercepted and are underestimated by parents, who are not aware of the serious consequences that can occur to permanent teeth. Dental emergencies, as explained in the Guidelines of the International Association of Dental Traumatology (IADT) [10], induce anxiety and distress in young patients, and in their parents too [10]. Furthermore, this experience often represents a child’s first dental examination [10]. All these factors determine an important degree of difficulty for the dental team [10]. In addition, extrusive luxation traumas, in addition to root and alveolar fractures can cause very painful occlusal interferences; therefore, they must be promptly treated [10]. So, the importance of knowing how to manage such an emergency is obvious. Often these traumatic events lead to tooth extraction and, therefore, serious problems with the eruption and alignment of the permanent teeth [11]. Therefore, dentists play an essential role in deciduous TDI treatment. The maxillary central incisors are the teeth most frequently involved, with an incidence as high as 80% [12]. Trauma to the supporting tissue is common. The most frequent injuries are extrusion, lateral luxation, and intrusion. Root fracture, on the other hand, is rarer, with an incidence of around 2% [13]. It is caused by severe trauma in an area of the crown close to the gumline, or even in correspondence with the alveolar bone [14].

The modified classification of root fractures is based on the location of the fracture line and is divided into [15]: the fracture of the apical third, the middle third, or the coronal third, and further subdivided into subcrestal and supracrestal.

Symptoms can range from mild to severe, and differential diagnosis includes extrusive tooth luxation.

Clinical examination sometimes shows a swelling of the alveolar region in the vestibular area with increased mobility of the affected elements.

Intraoral radiographic examination is essential and highlights the root fracture as a horizontal radiolucency line (if the inclination of the rays is parallel to the course of the fracture)**,** while an ellipsoidal radiolucency image appears if, as most frequently happens, the ray impacts the fracture at an angle, thus showing the double image of the vestibular and palatal sides. The differential diagnosis includes extrusive luxation, from which it differs radiographically because of the empty or radiolucent aspect of the apical and lateral portion of the alveolus [16]. Furthermore, there is often a lengthening of the periodontal ligament and the absence of fracture lines [17].

As far as therapy is concerned, most authors support the need to extract deciduous teeth that have suffered root fractures, especially type 3, possibly leaving in place the apical fragment, which will undergo a physiological process of rizolysis [18]. Sometimes, conservative therapy can be used for root fractures located in the middle or apical thirds, which are the most frequent. In these cases, splinting and occlusal stabilization should be performed. Healing occurs thanks to the interposition of connective tissue, despite the presence of mobility at the level of the fracture line [19]. According to Andreasen et al., the conservative choice was found to present a good percentage of success as early as 1998, considering the difficulty of the execution of splinting and the poor cooperation of pediatric patients [20]. Follow-up requires careful clinical and radiographic exams for the evaluation of the periodontal and pulp status. Particularly, attention to the onset and development of pulp canal obliteration (PCO) and pulp necrosis (PN) following TDI is required. PCO is characterized by the gradual, progressive and excessive deposition of tertiary dentin within the canal walls, visible radiographically as a qualitative reduction in the endodontic space [21].

The main purpose of this study was to conduct a scoping review of the scientific literature to evaluate the diagnostic–therapeutic process of root fractures in primary teeth, and then to establish the correct radiographic and clinical diagnostic tests and the therapeutic interventions to be performed. These were evaluated and analyzed for: the presence of further trauma; possible contextual dislocation; time elapsed between trauma and treatment; compliance with IADT guidelines; possible presence of the reabsorption of the apical portion of the root; the state of pulp vitality; and, finally, the duration of the follow-up.

## 2. Materials and Methods

The review was carried out by applying the “Scoping Review” method described by O’Malley and Arksey [22], and recently again discussed by Zachary Munn et al. [23]. Basically, it can be divided into the following phases: the identification of the research question; the search for relevant studies; the inclusion of studies; and the collection and synthesis of the results. Consequently, a working protocol was drawn up in accordance with the PRISMA (Preferred Reporting Items for Systematic Reviews and Meta-Analyzes) guidelines, dating from 2015 [24].

### 2.1. Research Questions

The main purpose of this study was to conduct a scoping review of the scientific literature to evaluate the diagnostic–therapeutic process of primary tooth fractures, and then to establish the correct radiographic and clinical diagnostic tests and therapeutic interventions to be performed. The following points were assessed: (1) age of the patient; (2) localization of the root fracture; (3) any intraoral diagnostic tests performed (radiographs, sensitivity tests to heat and/or cold and/or EPT, inspection, palpation, percussion); (4) any dislocation suffered (intrusive, extrusive or lateral); (5) the presence of resorption of the apical portion of the root (if so, in what time), diagnosable thanks to follow-up radiographic examinations; (6) emergency treatments performed, such as repositioning and stabilization techniques (type of splinting technique) or tooth extraction, and their compliance with IADT guidelines; (7) the maintenance of the crown without infectious or pulpal inflammatory phenomena or the development of PCO; (8) recommendations for maintenance after the trauma; (9) and follow-up.

The search strategy was performed in accordance with the PICO method [25], articulated in the following points:

(1) Population. We exclusively considered studies carried out on patients with primary dentition with one or more injured teeth in the incisal group affected by root fracture and/or dislocation (intrusive, extrusive, or lateral). Furthermore, we excluded root fractures of the deciduous molars.

(2) Intervention. All emergency therapeutic interventions were considered.

(3) Comparison. We compared the age of onset and the possible involvement of permanent tooth germs in patients with dislocation and root fracture injuries.

(4) Results. We considered the number of root fractures, the therapy, and the eventual healing during the follow-up. Only articles in English were selected, with no restrictions on publication date. Observational studies, randomized clinical trials, case reports and case series were included. Editorials, reviews, in vitro or animal studies, lecture abstracts, comments and letters were excluded.

### 2.2. Selection Criteria

**Figure 1:**
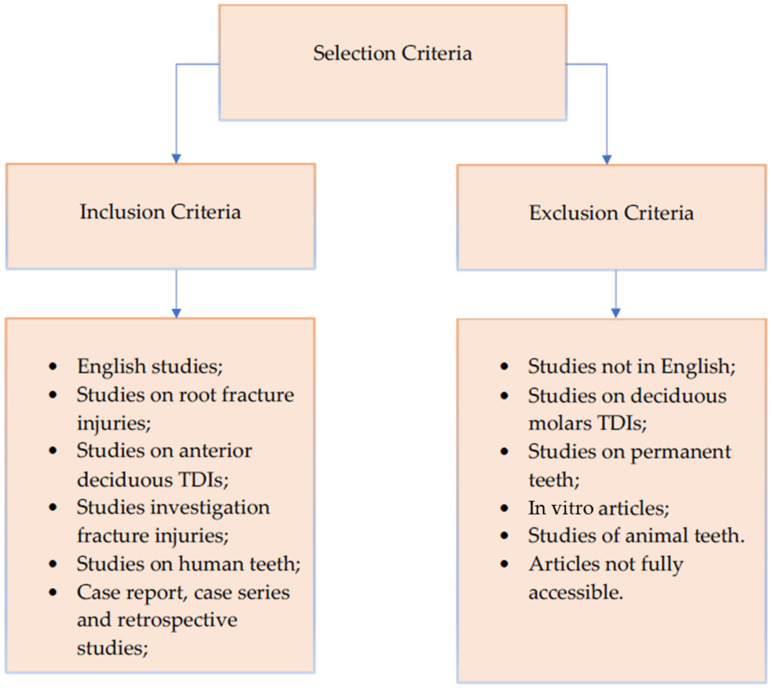
Diagram showing the inclusion and exclusion criteria used for this review.

The inclusion criteria are summarized in Figure 1.

### 2.3. Search Strategy

This study was carried out from June 2021 to August 2021, using the following databases: SCOPUS, Web of Science and finally MEDLINE/PubMed. The following keywords were chosen: ((root fracture) AND (tooth OR primary teeth)), ((root fracture) AND (tooth OR deciduous teeth)), (root fracture) AND (permanent germs). Two reviewers (ES and MSM) independently assessed and selected valid studies based on the titles and abstracts. They later worked together to select a final list of studies. When any disagreement arose, a third reviewer (NZ) resolved the question.

### 2.4. Study Quality Assessment

Three reviewers (ES, MSM and NZ) independently assessed the quality of the articles collected. To assess the level of agreement between the reviewers, the Cohen kappa coefficient was calculated and found to be moderate (value equal to 0.60) [26]. All disagreements that emerged were assessed and resolved objectively and in agreement by all three reviewers.

### 2.5. Data Extraction

The two lead reviewers (ES and MSM) gathered the following information from each study: the year of publication, the first authors, the age and gender of the affected patient, the number of samples and root fractures, the traumatized tooth, the location of the root fracture (apical third, middle third or coronal third), any other trauma (e.g., luxation), the maturation phase of the permanent incisor germs, the time range after trauma detected, the emergency interventions, and finally the duration of the follow-up. The data extracted from the selected articles were entered into a Microsoft Excel spreadsheet for final analysis.

## 3. Results

### 3.1. General Data of the Selected Patients

**Figure 2:**
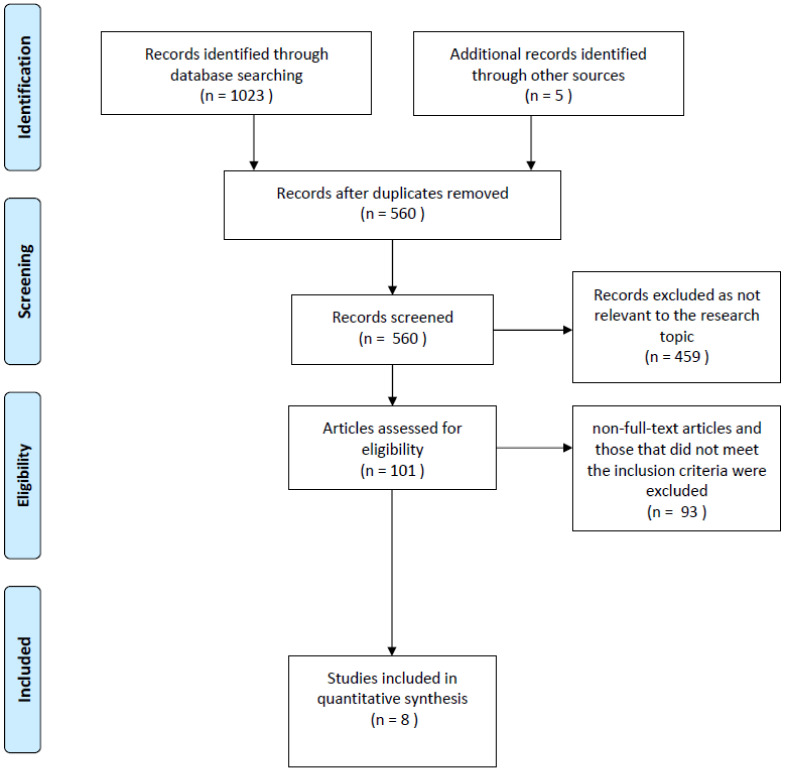
Flowchart of the literature search and selection process.

Figure 2 shows the flowchart and search strategy used to select the articles included in the final scoping review. Only eight articles fully met our inclusion criteria. Seven studies included case reports and only one was a large sample retrospective study, as shown in Table 1. The number of cases described in the 8 included studies was 46, with a total of 62 root fractures: 9 root fractures in the case reports, and 53 in the retrospective study. Patients’ ages ranged from 3 to 4 years. Regarding gender, 12 were girls and 34 were boys. One retrospective study included in this review reported the range rather than the patients’ mean age. Most children had suffered trauma from accidental falls while playing at home, while only one accident occurred at school. All the studies included in our review treated root fractures in the maxillary upper incisors. In one case, there was a root fracture of both maxillary incisors (Table 1).

### 3.2. Type of Root Fracture

In the case reports, six fractures lines were in the apical third [14,18,27,28,29,30], and three in the middle third [18,28,32]. As for the 53 teeth of the retrospective study, Cho et al. did not specify the exact location of the fracture, but wrote that all were fractures affecting the apex or the middle third of the root [31].

### 3.3. Further Trauma and/or Dislocation Suffered

Of the cases reported, lip oedema and bleeding were the most frequently reported facial injuries (*n* = 5); in two cases no facial lesions were found. Mobility was reported in four cases; specifically, this was of grade II in the two cases described by Kumari et al. [28] and in the case of Liu et al. [18]. Mobility was also highlighted by Brudza-Zwiech et al. in their root fracture case, but this time without specifying the degree [29]. Regarding dental luxation trauma, Bonanato et al. described a case with an extrusive vestibular luxation [14], Liu et al. [18] described a 1 mm palatal extrusive luxation, and Di Giorgio et al. noted a 3 mm one [30]. In the retrospective study by Cho et al., it was not specified whether the root fractures also involved luxation trauma [31].

### 3.4. Time to Treatment

Most of the included cases (*n* = 20) were treated within 24 h, while one case was treated 3 days after the event. In the study by Cho et al., 19 patients with root fractures were treated 24 h later [31]. Only the study by Flores et al. did not specify time to treatment exactly [32].

### 3.5. Intraoral Diagnostic Tests Performed

In all cases, an initial intraoral periapical radiograph was performed; this was decisive for the diagnosis and was repeated several times during the follow-ups. On average, about three intraoral radiographs were taken for each case. The studies with the most (*n* = 5) were those of Liu [18] and Bruzda-Zwiech et al. [29]. Most of the studies also evaluated the positivity of the percussion of the fractured primary teeth at the time of the trauma and the negativity during the check-ups.

### 3.6. Treatment

Out of a total of 62 fractures, the most performed treatment was splinting (*n* = 39) for a period ranging from 3 weeks to 3 months (with an average of six weeks). No treatment was performed in 23 root fractures. As for the case reports, seven fractures were treated with splinting and two were not splinted. In the study by Cho et al., there was no treatment for 20 of the fractures but 33 were splinted [31]. The splinting type mostly applied in the included cases was semi-rigid, held in place thanks to a composite resin material. In one study only, an orthodontic splint using brackets and 0,5 mm stainless steel wire was used [18]. On the other hand, Di Giorgio et al. preferred to use a flexible splint [30]. Most of the emergency treatments reported were conducted according to IADT guidelines.

### 3.7. Follow-Up

Follow-up ranged from a minimum of 4 months to a maximum of 3.5 years. Five teeth with a total of five root fracture lines were followed up to the eruption of the permanent incisor, all without sequelae. On average, in terms of frequency of follow-up, the second appointment took place 2–4 weeks after the first examination. After the second check-up, the follow-ups took place after longer intervals (6 months and 1 year, on average) in conformity with the IADT guidelines.

## 4. Discussion

Root fracture in primary dentition is relatively rare. It occurs more frequently in patients aged between 3 and 4 years, as the process of physiological rhizolysis begins [28]. As seen in the cases included in this scoping review, the etiology of TDI is commonly due to the incoordination of the growth age (1 to 3 years old), resulting from accidental falls during play, either at home or during sporting activities [28]. As evidenced by the literature, the most commonly involved teeth are the maxillary central incisors [10].

The treatment of primary root fractures represents a great challenge for dentists due to diagnostic and therapeutic difficulties; furthermore, domestic, and emotional management of TDIs may cause trouble for the entire family. The degree of cooperation of the child plays an essential role during conservative treatment [29]. In accordance with the recommended IADT guidelines, all cases included in this scoping review underwent periapical radiographs for diagnostic purposes [10]. In accordance with the literature, all root fractures were detected in the middle third of the root or, more commonly, the apical third. We can affirm that most cases included in the review complied with the IADT guidelines because most of the primary teeth that were fractured and mobile or affected by luxation trauma were splinted and followed over time. Splinting makes it possible to protect the tooth from further damage and protect the supporting tissue of the tooth in order to allow the periodontal fibers to regenerate [33]. In support of treatment, a systematic review of the literature by Andreasen et al. also showed that the TDIs to the primary teeth must be treated quickly to avoid further damage [34]. Only in 23 fractures out of 62 was the splinting of traumatized dental elements not performed [27,29]. In the first study, by Gadicherla et al., this was in accordance with the IADT guidelines: the affected tooth had no mobility, so it was not splinted [27]. However, the case of Bruzda-Zwiech et al. did not conform to these guidelines because, despite mobility, no treatment was carried out [29]. The type of splint mostly commonly applied in the included cases was a semi-rigid splint, held in place thanks to a composite resin material [14,28,30,32]. Only in one study was an orthodontic splint using brackets and 0,5 mm stainless steel wire used [18]. As highlighted by Liu et al. [18], this splinting method has the advantage of being more easily managed in very young patients, because it is easier to position and makes it possible to evaluate the mobility of the traumatized tooth without removing the brackets [18]. In the retrospective study by Cho et al., 182 luxated primary teeth were examined, of which 90 had been treated with semi-rigid splinting (flexible round stainless steel wire). The results showed that the splinting root of fractures gave a good prognosis. Particularly, the splinted group was 4.67 times more likely to have clinical success than the observation group, consisting of non-splinted teeth with root fractures [31]. In addition, the splinting and the consequent stabilization of the affected tooth makes it possible to prevent the child from suffering further trauma. Moreover, splints should allow some functional movement of the traumatized teeth [35]. It has been widely demonstrated in the literature that rigid splinting determines immobilization of the affected teeth, with a greater risk of developing ankylosis [35]. On the other hand, an elastic-type splint was associated with higher healing rates [35]. By elastic/flexible splinting we mean stainless steel wires with a diameter not exceeding 0.3–0.4 mm, associated with composite resin. This type of splinting has numerous advantages: easy application, especially in the case of non-cooperative pediatric patients; easy maintenance of oral hygiene; and minimum physical encumbrance of aesthetic, phonatory and masticatory function. Furthermore, it does not invade the soft tissue, which is often swollen from the trauma, and does not cause interference for vitality tests and/or other dental procedures [35]. As for the splinting time interval, variable times were found, with more values above the average of those in the IADT guidelines [14,18,28]. The last IADT guidelines recommend flexible splinting for 4 weeks. If the root fracture is close to the cervical area of the tooth, splinting stabilization is recommended for up to 4 months [10].

Regarding the possible treatment of teeth with root fractures, none of the cases included in our scoping review underwent pulpotomy or pulpectomy therapy. Furthermore, no periapical lesions were found in the intraoral radiographs of the cases examined. Only in the study by Di Giorgio et al., at the radiographic follow-up one year after the trauma, was a PCO not associated with coronal dyschromia documented [30]. PCO is characterized by the gradual, progressive and excessive deposition of tertiary dentin within the canal walls, radiographically seen as a qualitative reduction in the endodontic space [36]. In the literature, as evidenced by Santos et al., its prevalence varies significantly, with percentages ranging from 3 to 48% [21]. A retrospective study of 112 primary maxillary incisors with a 9-year follow-up found that PCO developed in 54% of traumas, and within 12 months of the event [21]. Furthermore, there was no association between PCO and secondary pulp necrosis [21]. Therefore, the treated cases included in this scoping review were not in line with the literature because none of the authors, with a single exception, mentioned a developed PCO. This could be due to a lack of diagnosis of PCO, to inadequate X-ray follow-up during the subsequent years, or because the development of PCO did not enter the inclusion criteria chosen for these studies.

Disinfection of the traumatized site, proper maintenance of oral hygiene, and an adequate diet all play an important role in the healing process of traumatized teeth [37]. In numerous studies, rinsing with chlorhexidine-based mouthwashes [18,27,29] or topical application using soft brushes has been recommended [30]. Finally, a soft diet was indicated in all cases to prevent further trauma to the affected teeth. For this reason, it is necessary to instruct the child’s parents in a concise and clear manner so that they cooperate, to promote healing.

Regarding the follow-up and outcomes, resorption of the apical root fragment occurred in all but two of the cases included in this scoping review. This type of resorption of the fractured root apex takes place in a relatively short period of time (after 3 months, as reported by Liu et al. [18]). A working hypothesis is that the fracture trauma triggers the activation of osteoclasts, with mechanisms not yet fully understood [38,39]. However, according to Nam et al., resorption of the apical fragment of primary teeth is a form of atypical resorption (ARR) [40]. The latter is distinguished by a circumferential peripheral resorption pattern along the lateral and/or apical root surface of a primary maxillary incisor [40]. Therefore, the diagnosis is essentially radiological. Atypical resorption of the root is distinguished from inflammatory resorption because it is accompanied by periapical bone radiolucency; moreover, the root takes on a typical bowl-like appearance. No clinical cases included in our scoping review reported any sequelae to the germs of the permanent teeth, in accordance with the data found in literature [10,41]. The absence of damage to the germ of the permanent tooth occurs thanks to the apical root fragment, which stands in its original position while the remaining coronal fragment undergoes a dislocation to the periodontal ligament and the nerve bundle as a result of the trauma [42]. Therefore, the repositioning and splinting of the coronal fragment should not injure the permanent germ [42]. Hence, the conservative treatment of primary root fractures is highly beneficial. To support this, a Brazilian retrospective study looked at the primary TDI treatments of 351 children between the ages of 1 and 4 [43]. The authors stressed that careful monitoring is the preferred therapeutic choice for TDI in primary dentition, even in its most severe forms [43].

Ultimately, the findings of this scoping review prompt several interesting clinical considerations:

1. The root fracture of primary teeth is a rare injury predominantly found in the upper incisors of children aged 3 to 4 which, if not managed properly, can cause numerous complications, including premature tooth loss and altered eruption of the permanent teeth, as well as possible psychological repercussions.
2. During the first diagnostic check, we would emphasize the need for greater attention to the interpretation of the radiographic images regarding the early resorption of the apical root, which generally should be interpreted as a physiological response.
3. Regarding treatment, repositioning and splinting of the coronal fragment does not damage the permanent germ [42]. As explained above, this occurs because the apical fragment remains in the same position. So, the conservative treatment of the root fractures of primary teeth is highly beneficial. Tooth repositioning and semi-rigid splinting therapy could be conservative methods to avoid extraction in pediatric patients.
4. Manual repositioning of the dislocated coronal fragment and subsequent splinting immediately after the trauma gave positive long-term results. The use of orthodontic splinting techniques is poorly described in the literature, but this could be a useful method when manual tooth repositioning is difficult and should therefore be further investigated.
5. PCO of the crown of tooth affected by the root fracture is frequent and should not be understood as a pathological phenomenon. This response from the dental pulp is often underestimated, or not properly investigated in clinical studies.

## 5. Conclusions

In this scoping review, the authors affirm that most of the initial motivations were achieved, even though some of the studies included did not completely analyze all the outlined research objectives. There was little uniformity in the collection of data. For this reason, we would recommend making the diagnostic therapeutic process more homogeneous, with particular attention to radiographic exams. It is necessary to carry out further clinical studies with large samples and adequate data collection to be able to produce satisfactory systematic reviews and to produce metanalysis of the therapeutic options considered in this scoping review.

## Figures and Tables

**Table 1:** Data from the eight articles included in the review. Legend: AT = apical third; MD = middle third; IPR = intraoral periapical radiograph; M = male; F = female.

Author and Year	Type of Article	N◦ of Samples	N◦ of Dental Fracture	Traumatized Tooth	Localization of Fracture	Any Other Trauma	Permanent Germs Maturation Stage	Tests Performed	Range Time after the Detected Trauma	Age	Gender	Therapeutic Intervention	Follow-Up	Outcome
Bonanato et al., 2009 [14]	Case report	1	1	61	AT	Extrusive luxation vestibularly	ns	IPR	1 day	3	F	Repositioning and splint (semi-rigid containment with a 0.5 orthodontic wire affixed with photopolymerizable resin) for 3 weeks	12 months	Permanence of the apical fragment and reduction in the separation between the root fragments, no discoloration
Liu et al., 2013 [18]	Case report	1	2	51 61	AT MD	Grade II mobility Gum bleeding Extrusive Luxation of 1 mm palatally Grade II mobility Gum bleeding	Crown 3/4 complete	IPR	Same day	3.5	F	Splint (orthodontic brackets and 0.5 mm stainless steel wire) for 3 months. Repositioning and splint (orthodontic brackets and 0.5 mm stainless steel wire) for 3 months	2.5 years	After 3 months the root resorption of both apical fragments was almost completed
Gadicherla et al., 2016 [27]	Case report	1	1	51	AT	No mobility	Crown 3/4 complete	IPR	3 days	3.5	F	Any treatment	4 months	No complications
Kumari et al., 2017 [28]	Case report	2	1 1	51 51	AT MD	Grade II mobility Grade II mobility	Crown 3/4 complete	IPR	Same day Same day	4 4	M M	Splint (semi rigid wire-composite splint) for 2 months. Splint (semi-rigid wire composite) for 4 weeks	36 months 24 months	Resorption of the apical fragment and apical root after 2.5 years. Resorption of the apical fragment and apical root after 1 year
Bruzda-Zwiech et al., 2018 [29]	Case report	1	1	61	AT	Mobility	ns	IPR	Same day	3	M	Any treatment	3.5 years	At the 8-month follow-up there was evidence of healing of the root fracture with calcified tissue, at 3.5 years there was a complete resorption of the apical fragment and of the entire root
Di Giorgio et al., 2021 [30]	Case report	1	1	51	AT	Extrusive luxation of 3 mm	ns	IPR	Same day	3,5	M	Repositioning and splint (orthodontic flexible splint) for four weeks	3 years	After 1 year there was pulp canal obliteration and resorption of the apical fragment
Cho et al., 2017 [31]	Retrospective study	38	53	ns	ns	ns	ns	ns	<24 h 14 >24 h 19	ns	30 M 8 F	Any treatment: 20 Splinting: 33	6 months	Any treatment: failure 14 Splinting: failure 11

## Data Availability

The data supporting the findings of this scoping review are available within the article.
